# The Mediational Role of Burden and Perceived Stress in Subjective Memory Complaints in Informal Cancer Caregivers

**DOI:** 10.3390/ijerph17072190

**Published:** 2020-03-25

**Authors:** Marta Ramos-Campos, Rosa Redolat, Patricia Mesa-Gresa

**Affiliations:** 1 Junta asociada provincial de Valencia de la Asociación Española contra el Cáncer, 46010 Valencia, Spain; Marta.Ramos@aecc.es; 2 Psychobiology Department, Universitat de València, 46010 Valencia, Spain; Rosa.Redolat@uv.es

**Keywords:** cancer caregivers, burden, memory complaints, perceived stress, resilience, self-esteem

## Abstract

The role of informal caregiver of cancer patients is considered a situation of chronic stress that could have impact on cognitive functioning. Our aim was to evaluate differences in perceived stress, subjective memory complaints, self-esteem, and resilience between caregivers and non-caregivers, as well as the possible mediational role of burden in caregivers. The sample was composed of 60 participants divided into two groups: (1) Primary informal caregivers of a relative with cancer (CCG) (*n* = 34); and (2) non-caregiver control subjects (Non-CG) (*n* = 26). All participants were evaluated through a battery of tests: Socio-demographic questionnaire, subjective memory complaints questionnaire (MFE-30), Rosenberg Self-Esteem Scale, resilience (CD-RISC-10), and perceived stress scale (PSS). The CCG group also completed the Zarit burden interview. Results indicated that CCG displayed higher scores than Non-CG in MFE-30 (*p* = 0.000) and PSS (*p* = 0.005). In the CCG group, Pearson correlations indicated that PSS showed a negative relationship with resilience (*p* = 0.000) and self-esteem (*p* = 0.002) and positive correlation with caregiver’s burden (*p* = 0.015). In conclusion, CCG displayed higher number of subjective memory complaints and higher perceived stress than Non-CG, whereas no significant differences were obtained on self-esteem and resilience. These results could aid in designing new intervention strategies aimed to diminish stress, burden, or cognitive effects in informal caregivers of cancer patients.

## 1. Introduction

Cancer is a disease that exerts a great psychological impact on patients and those around them [1,2]. Family members and people close to cancer patients also suffer their own adaptation process with many emotional and functional disturbances in their daily lives. The role of the informal caregiver has been related to a situation of chronic stress that produces harmful effects on physical and mental health [3,4] and can be associated with cognitive impairment [5]. Different studies have observed that caregivers display higher rates of physical and psychological problems [3] and higher incidence of anxiety, depression, and hospitalizations than the general population [6,7,8]. These consequences have been observed in diverse samples [7,9]. Current research has also addressed some positive aspects associated with the task of taking care of cancer patients, including effects as gains or benefits related to the caregiving task [10,11,12,13].

Taking into account the impact of stress on health, physical, and mental functioning, the consequences of caregiving could be modulated by various variables. Some of them involve the patients (characteristics, type and severity of their condition, prognosis of disease, etc.) [14,15], while others affect the caregiver, such as certain personality traits [16,17], coping ability [6,18], or burden levels [19]. Regarding risk factors for the caregiver, studies have highlighted insufficient social support [20,21] or low perceived social support [22,23], low resilience [24,25], and low self-esteem [26]. 

During the disease progress, caregivers must assume different roles at physical, economic, social, and mental levels [27,28]. Deterioration associated with the caring task could be manifested as an impairment of memory (both immediate and delayed), reduced verbal fluency, vocabulary and executive functions decline [5,29] and, in some subjects, as an increased risk of developing dementia [30,31]. In fact, different studies have shown that caregivers of people with dementia are more vulnerable to suffer cognitive impairment [30,32,33]. Physical consequences and fatigue can influence cognitive abilities such as concentration, which is remarkably decreased in caregivers [34]. In addition, a poorer metabolic health accelerates aging, thereby increasing vulnerability to cognitive impairment [32]. 

Although different studies have shown some of the consequences of caregiving on the cognitive health of caregivers, few studies have analyzed in detail their subjective memory complaints. Vitaliano et al. [33] observed that caregivers of people with Alzheimer’s disease (AD) reported more subjective cognitive problems than non-caregivers. A recent systematic review addressing neuropsychological consequences of the stress associated to the caregiving task indicated that caregivers have shown alterations in cognitive functions including memory, attention, inhibitory abilities, and speed processing, although results are heterogeneous. In addition, these dysfunctions may become more pronounced the more the caregiving task is prolonged [35].

Taking into account these questions, the main purpose of this study was to assess and describe, using a quasi-experimental approach, the consequences of exposure to the chronic stress produced by the task of continuous care, for the health of cancer patients’ caregivers. Specifically, we have studied the consequences of the burden induced by the caregiving situation on subjective memory complaints and their relationship with other variables, including perceived stress, personal resilience, and self-esteem, taking into account the potential influence of modulating variables such as age, gender, and disease prognosis on the subject.

## 2. Materials and Methods 

### 2.1. Participants

A total number of 60 subjects participated in the present study. We performed a quasi-experimental research study comparing scores between two groups: (1) Primary informal caregivers of a relative with cancer (CCG) (*n* = 34, 18 women and 16 men, mean age = 50.05 years); and (2) non-caregiver control subjects (Non-CG) (*n* = 26, 16 women and 10 men, mean age = 47.46 years). CCG and their relatives invited to participate in the study were contacted through a Spanish association called ‘*Asociación Española contra el Cáncer*’ that provides assistance to cancer patients and their families in various hospitals in the area. It is a nonprofit association that operates nationwide and has offices in all the provinces of Spain. Specifically, this study was conducted with the caregivers of cancer patients attending the office in the province of Valencia. The CCG group was formed by primary caregivers of a relative with a cancer diagnosis voluntarily recruited from different hospitals and health institutions of Valencian Community (*Hospital Clínico Universitario of Valencia, Hospital de la Ribera of Alzira and Hospital of Sagunto*). Inclusion/exclusion criteria for CCG were: Being a primary informal caregiver of a patient with diagnosis of any type of cancer at any stage of the disease, not being a professional caregiver (not getting paid for the task of care), and not suffering from chronic diseases. All GCC participants reported providing at least 8 h of daily care to the cancer patient and for at least three months at the time of completion of the questionnaire battery.

The Non-CG group was composed of volunteer participants displaying similar socio-demographic characteristics to the caregiver group. These subjects were voluntarily recruited, through posters and advertisements, in hospitals, health institutions, and the University of the Valencian Community. Eligible Non-CG matched the gender and age distribution of the CCG and had no previous experience as primary caregiver of a dependent person with a chronic disease during their life.

### 2.2. Measures

Different instruments were employed in this study and a variety of measures were obtained: (1) Participants’ socio-demographic data, personal resources in terms of resilience, self-esteem, cognitive decline/status through their reported subjective memory complaints, and other data of interest (CCG and Non-CG groups); and (2) caregivers’ evaluation of the caregiving situation in terms of levels of burden and perceived stress (only CCG group).

#### 2.2.1. Socio-Demographic Questionnaire

A socio-demographic questionnaire was designed ad hoc in order to record relevant information about the participants. The questionnaire was composed of two sections. The first one recorded general data about all the participants (CCG and Non-CG groups) such as gender, occupation, marital status, kinship, educational level, possible sleep problems, physical, and leisure activities, as well as if they feel tired when they perform activities that are part of daily routine (fatigue), etc. The second section was completed only by caregivers (CG group) and it was aimed to collect specific data on care-recipients’ characteristics, as well as illness-related information of the cancer patients (regarding type of cancer, prognosis, treatment, time since diagnosis).

#### 2.2.2. Memory Failures of Everyday (MFE-30)

The Memory Failures of Everyday (MFE-30) is the Spanish adaptation of the original questionnaire called Memory Failures of Everyday Questionnaire (MFE) [36]. The scale was validated by Lozoya-Delgado et al. [37] and displays good psychometric properties (Cronbach’s alpha = 0.93) and a one-dimensional structure. This test was designed to measure subjective memory failures of daily living and is composed of a 5-point Likert scale with 30 items, ranging from 0 (never or almost never) to 4 (always or almost always) (range: 0–120 scores).

#### 2.2.3. The Connor-Davidson Resilience Scale (CD-RISC-10)

The Connor-Davidson Resilience Scale (CD-RISC-10) [38] was used in order to measure participants’ resilience. The Spanish version of this scale, adapted by Serrano-Parra et al. [39], has been well-validated and displays a one-factorial structure and high internal consistency (Cronbach’s alpha = 0.81). It is composed of 10 items coded on a 5-point Likert scale, ranging from 0 (absolutely) to 4 (almost always) (range: 0–40 points).

#### 2.2.4. Rosenberg Self-Esteem Scale

The Spanish version of the Rosenberg Self-Esteem Scale [40], adapted by Atienza [41], was employed to measure participants’ global self-esteem. The scale has been well-validated [42] and shows good psychometric properties (Cronbach’s alpha = 0.87). It is composed of 10 items with a 4-point Likert scale, ranging from 1 (totally disagree) to 4 (totally agree) (range: 0–40 scores).

#### 2.2.5. Perceived Stress Scale (PSS)

The Perceived Stress Scale (PSS) [43] was designed to measure the level of perceived stress reported by the respondents over the last month. The Spanish adaptation by Remor [44], with a Cronbach’s alpha = 0.86, was used in this study. This scale is composed of 14 items in which respondents have to answer the extent to which life situations are perceived as stressful. It is rated on a 5-point Likert scale, ranging from 0 (never) to 4 (very often) (range: 0–56 scores).

#### 2.2.6. Zarit Caregiver Burden Interview (ZCBI)

The level of burden experienced by caregivers was measured through the Spanish version of the Zarit Caregiver Burden Interview (ZCBI) [45], adapted by Marín et al. [46]. These instruments show good psychometric properties (Cronbach’s alpha = 0.91). The ZCBI measures the level of subjective burden experienced by caregivers by means of questions regarding caregiver’s perceptions over taking care of their relative. It is composed of 22 items coded on a 5-point Likert scale, ranging from 0 (never) to 4 (nearly always) (range: 0–88 points). Scores obtained were classified into three levels of burden corresponding to the following cutoffs: Absence of burden (scores from 22 to 46), slight burden (scores from 47 to 55), and intense burden (scores from 56 to 84).

### 2.3. Procedure

Candidate participants were recruited from diverse hospitals and health institutions and were informed of the purpose of the study. After accepting their voluntary participation and giving their signed informed consent, subjects were provided with a pack of self-administered questionnaire which they had to give back to the researchers or leave in the nursing control station for further analysis. All participants received a briefing from the researchers on how to complete the battery of questionnaires. In order to avoid social desirability bias as far as possible, participants were guaranteed anonymous and confidential contribution. This study was approved by the Ethical Research Committee of the University of Valencia and complies with the regional, national, and European laws governing the use of human subjects in research (Declaration of Helsinki, Seventh revision, 2013).

### 2.4. Statistical Analyses

Data was analyzed with the Statistical Package for Social Sciences (SPSS, IBM) for Windows (version 26.0, Madrid, Spain). In order to obtain the differences in the scores obtained in the assessment instruments between CCG and Non-CG groups, the Student t-test for independent samples were carried out. Moreover, there have also been bivariate correlations between the variables “Memory”, “Resilience”, “Self-esteem”, “Stress”, and “Burden” into the CCG group. Regarding the evaluation of the possible relationships between the variables obtained, as well as the modulatory role of burden and perceived stress in caregivers, bivariate Pearson correlations and multivariate regressions were also carried out in CCG group. The criteria of normality and homoscedasticity necessary to perform parametric tests are met. All data are presented as mean ± standard error of the mean (SEM). In all cases, the level of significance was set at *p* < 0.05.

## 3. Results

### 3.1. Socio-Demographic Characteristics of the Participants

The full sample was composed of 60 participants, 34 women (56.7%) and 26 men (43.3%) aged 48.95 ± 1.64 years (range: 19–79). A t-test was performed with the age variable, and it was observed that there were no significant differences between the GCC and Non-CG group for this variable. At the beginning of the study, CCG was composed of 51 eligible caregivers approached for participation in the study, but then, five refused to participate and 17 did not give the questionnaire pack back. The reasons for refusing to participate in the GCC group were lack of time or deterioration in the health condition of the patient being treated. Finally, CCG was therefore comprised of a total of 34 participants (participation rate 66.67%), 16 men (47.1%), and 18 women (52.9%) aged 50.08 ± 13.94. Most of them lived with the cancer patient (88.2%), being their partner (73.5%), sons or daughters (14.7%), parents (5.9%), or brothers/sisters (5.9%). Cancer patients were aged between 52.41 ± 1.86 years, with 61.8% of women and 38.2% of men. At the time of the study, patients had been diagnosed for 3–6 months (32.4%), 6–9 months (5.9%), 9–12 months (5.9%), and more than 12 months (55.9%). Prognosis was favorable for most of them (58.8%), in comparison with those that obtained a non-favorable prognosis (41.2%).

For the Non-CG, a total of 26 out of 49 approached non-caregivers were finally included in the study (participation rate 53.06%). In this group, the reasons for the decrease in sample size were lack of time to participate in the study and failure to return completed questionnaires. Participants were more likely to be women (*n* = 16, 61.5%) rather than men (*n* = 10, 38.5%), aged 47.46 ± 2.15 and displayed similar socio-demographic characteristics to CCG except that they had not performed informal caregiving throughout their lifetime. Detailed CCG and Non-CG socio-demographic characteristics are shown in Table 1, and characteristics of cancer patients are included in Table 2.

### 3.2. Differences between Caregivers and Non-Caregivers in Psychological Questionnaires

**Figure 1:**
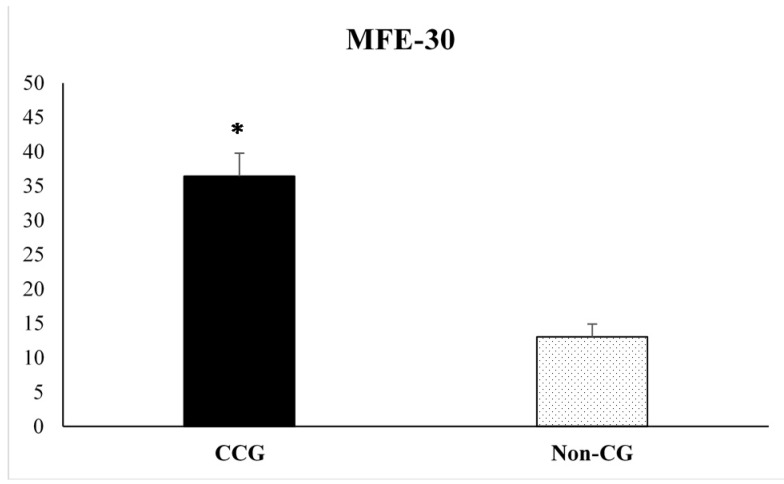
Scores obtained on the Memory Failures of Everyday scale (MFE-30) by the informal cancer caregiver’s group (CCG) and the non-caregivers control group (Non-CG). Data are presented as mean ± SEM. (*) *p* < 0.0001, CCG vs. Non-CG.

**Figure 2:**
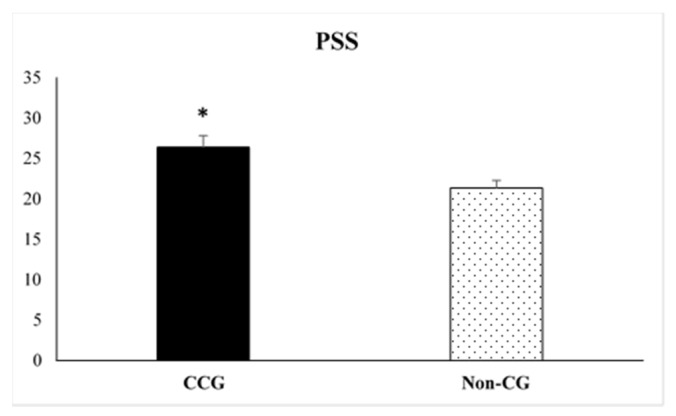
Scores obtained on the Perceived Stress Scale (PSS) by the informal cancer caregiver’s group (CCG) and the non-caregivers control group (Non-CG). Data are presented as mean ± SEM. (*) *p* < 0.01, CCG vs. Non-CG.

Student’s t-test for independent samples were analyzed in order to obtain possible significant differences between CCG and Non-CG on the variables assessed in the present study. Student’s t-test for independent samples revealed statistically significant differences between both groups on MFE-30 (t = 6.12, *p* = 0.0001) and PSS tests (t = 2.91, *p* = 0.005). CCG reported higher number of subjective memory complaints (36.44 ± 3.33) and higher levels of perceived stress (26.38 ± 1.45) compared to Non-CG (13.04 ± 1.87 and 21.35 ± 0.95, respectively) (see Figure 1 and Figure 2).

**Figure 3:**
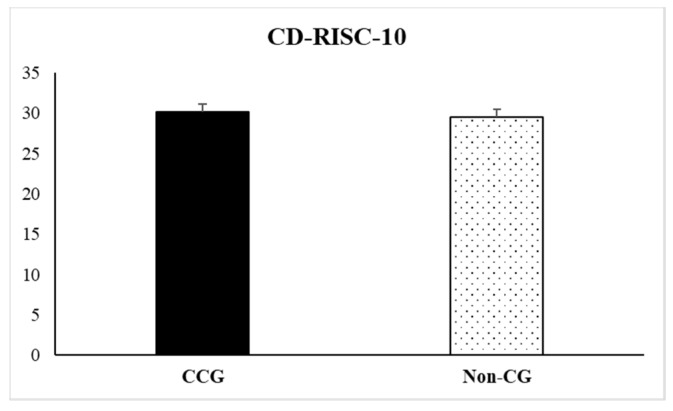
Scores obtained on the Connor-Davidson Resilience Scale (CD-RISC-10) by the informal cancer caregiver’s group (CCG) and the non-caregivers control group (Non-CG). Data are presented as mean ± SEM.

**Figure 4:**
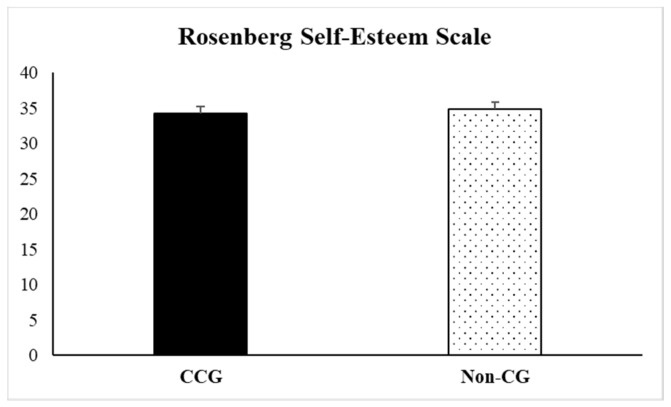
Scores obtained on the Spanish version of the Rosenberg Self-Esteem Scale by the informal cancer caregiver’s group (CCG) and the non-caregivers control group (Non-CG). Data are presented as mean ± SEM.

Regarding data obtained in the scales of CD-RISC-10 and self-esteem, no statistically significant differences were observed (t = 0.44, *p* = 0.66 and t = −0.51, *p* = 0.60, respectively). Nevertheless, descriptive data indicated that CCG showed higher scores than Non-CG (29.50 ± 0.99) on the resilience questionnaire (30.11 ± 0.97), not observing these differences on self-esteem measures (see Figure 3 and Figure 4).

Regarding gender-related differences, results indicated that there was a tendency of signification (*p* = 0.05) on PSS test. Descriptive data indicated that women showed higher perceived stress (28.78 ± 1.63) than men (23.68 ± 1.73) in the CCG group, while the opposite effect was observed in the non-caregiver group, i.e., men showed more perceived stress than women in the control group (22.60 ± 2.19 and 20.56 ± 1.73, respectively).

Analysis of variance (ANOVA) was also performed, in the CCG group only, in order to obtain possible differences based on the prognosis of the cancer patients. Significant differences were observed in the scores obtained in the PSS test (F (1.24) = 5.09, *p* = 0.04), showing higher perceived stress in those caregivers in whom the patient’s prognosis was “unfavorable” (48.43 ± 3.67) with respect to those who had a “favorable” prognosis (38.10 ± 2.84). The remaining measures in the questionnaires did not reach statistical significance for the prognosis variable.

### 3.3. Caregivers Burden and Subjective Memory Complaints: Correlations and Categorization of Participants on MFE-30 and ZCBI 

Bivariate Pearson correlations were performed in order to analyze possible relationships between the different measures obtained in the CCG group (see Table 3). Data obtained indicated that resilience showed a significant positive correlation with self-esteem (r = 0.410, *p* = 0.016) and negative correlations with perceived stress (r = −0.598, *p* = 0.000) and burden (r = −0.414, *p* = 0.015). Measures of self-esteem showed significant negative correlation with perceived stress (r = −0.502, *p* = 0.002). Finally, perceived stress displayed by CCG was positively correlated with burden in a moderate statistically significant way (r = 0.492, *p* = 0.003). Data related to subjective memory complaints did not show significant correlations with the other variables.

Taking into account scores about the levels of burden obtained in ZBI by CCG, data indicated that the average of the group indicated an absence of burden (42.35 ± 2.39). If we classify the caregivers into the different levels of burden obtained it was observed that 64.7% of caregivers showed no burden, 14.7% showed slight burden, and 20.6% indicated intense burden.

Regarding subjective memory complaints scores obtained in MFE-30 in the CCG, data were categorized into performance categories corresponding to the following scale: (1) Scores lower than 8 points indicated optimum performance, (2) scores between 8 to 35 points indicated normative performance with few significant failures, (3) scores between 36 to 50 points showed impaired performance with significant failures, and (4) scores equal to 50 points were indicative of serious impaired performance. Taking this classification into account, data obtained in CCG showed a percentage of 52.9% with normative cognitive performance with few significant failures, 29.4% showed impaired performance with significant failures, and a percentage of 17.6% showed serious impaired performance. On the other hand, 34.6% of Non-CG showed optimum performance and 65.4% of them showed normative performance with few significant failures.

### 3.4. Multivariate Regression Analysis between Data in Psychological Questionnaires of Caregivers, with Prognosis and Duration of the Illness of Cancer Patients 

Multivariate regression analyses showed that prognosis of the illness in cancer patients significantly predicted the level of burden of the caregivers (β = −0.371; *p* = 0.03), noting that the worse the cancer patient’s prognosis, the higher the level of perceived caregiver burden. In terms of duration of the illness of cancer patient’s prediction, the study results indicated that this variable positively predicted perceived stress displayed by CCG group (β = 0.45; *p* = 0.011).

## 4. Discussion

The main objective of the present study was to analyze effects of the care of oncological patients including subjective measures of cognitive performance. A comparison between informal caregivers of cancer patients and an age-matched sample of non-caregivers was performed taking into account different psychosocial measures (subjective memory complaints, perceived stress, resilience, and self-esteem). In order to explore the impact of the caregiving task more in depth, possible relationships between all variables were also analyzed. Furthermore, a detailed analysis of the scores obtained by the caregivers in the MFE-30 and ZCBI questionnaires was performed. Results indicated that CCG displayed a significantly higher number of subjective memory complaints and reported higher perceived stress than Non-CG. No statistically significant differences were found between CCG and Non-CG on self-esteem and resilience. Regarding the exploration of possible relationships between different variables analyzed in the CCG our data suggested that measures of resilience showed a positive relationship with self-esteem and a negative one with perceived stress and burden. Concerning the level of perceived stress by the CCG, there was a negative relationship with self-esteem and a positive one with burden. However, contrary to what was expected, no significant correlations were observed between subjective memory complaints and the rest of variables. Finally, more detailed analyses were carried out on the CCG group, observing that they presented higher levels of perceived stress when the prognosis of cancer patients was unfavorable. In addition, multivariate regressions were performed between socio-demographic data and psychological variables in caregivers. It was observed that the prognosis could predict the level of burden and the duration of the illness predicted the level of perceived stress in CCG group.

Prior research has suggested that during earlier stages of cognitive decline, subtle memory complaints may not have a functional impact, although it has been reported that poor results obtained in MFE-30 may be an indicator of future development of dementia [37,47]. Many studies directed to analyze the impact of caregiving on cognition have focused on the effects of taking care of people with dementia suggesting that caregivers display greater vulnerability to cognitive decline [32,48]. 

In our study, CCG displayed higher subjective memory complaints than Non-CG. These results are in line with those obtained in a recent study in which informal caregivers of oncologic patients reported alterations in a cognitive screening test [49] although results were modulated by patients’ characteristics and preferences. These consequences could be related to factors such as stress or health care and, in some cases, may be temporary [49]. In a previous review focused on analyzing different studies related to the effects of stress on cognition in parents of children with cancer, an important effect of this variable on cognitive tasks such as attention or working memory was observed. These effects are also related to the development of pathologies such as depression or anxiety associated with children’s illness [50]. We must take into account that in our study, the mean age of the CCG was 48.95 years and the mean age of the patients was 52.41 years and, therefore, results obtained could be difficult to compare with prior studies carried out with younger subjects. The questionnaire MFE-30 was employed in order to compare cognitive complaints between CCG and Non-CG. Higher scores in this test could indicate deficits not only in memory but also in attentional and executive mechanisms [37]. It is important to consider that attention constitutes an essential part of the care situation which may adversely affect cognition and, consequently, the health of caregivers and the quality of the care they provide [51]. 

Differences obtained in the measure of perceived stress are consistent with previous studies indicating that both caregivers of cancer patients [52] and of AD patients [5] displayed greater perceived stress than non-caregivers. Different studies have evaluated perceived stress in relation to resilience and/or severity of the care-recipient’s illness. Perceived stress usually is very high between parents of children with cancer disease who adopt different strategies for coping with stress [53]. Skok et al. [54] found that the level of stress perceived by mothers was more related to their well-being than to the severity of the illness of the child they were taking care of. In the current study, results suggest that caregivers of patients whose prognosis was worst reported higher levels of perceived stress than those who were caring for subjects whose prognosis was more favorable. Furthermore, the level of stress reported by caregivers was negatively correlated with resilience. Moreover, perceived stress can be modulated by the date of diagnosis of the oncologic patient. Those participants who have been caregiving for the patient for a period between nine and twelve months displayed higher stress than those who were caregiving for a period between three and six months, or more than a year. These results support prior research suggesting that time could contribute to the individual adaptation process and resilience [16,55]. For that reason, we need more information about what variables and experiences influence the transition towards the new role adopted for the caregivers of cancer patients who need to adopt new competences and responsibilities [56]. 

Regarding the modulatory role of different variables measured in our study, results obtained revealed a positive moderate correlation between self-esteem and resilience in caregivers, although no statistically significant differences between CCG and Non-CG were found. Previous studies have found higher self-esteem scores in caregivers of colon cancer patients [51]. One possible explanation may be that caregivers of these patients could be more involved in the care of their relatives and therefore perceive the caregiving task as a challenge that fosters greater personal growth. However, levels of self-esteem in cancer caregivers can be also associated with economic resources available [57]. 

Caregiver burden and its associated factors is one of the most addressed issues in the literature [19,58]. Previous research assessing the level of burden has been conducted mainly with caregivers of AD patients, for whom high levels of caregiver burden have been reported [59]. Other studies involving informal cancer caregivers have obtained burden levels ranging from low [60] to moderate or severe [61]. In our study, 64.7% of caregivers reported absence of burden while 14.7% reported slight burden and 20.6% of them intense burden. Among factors that may increase vulnerability to suffer burden in caregivers, being female, low education, and low social support have been identified [59]. Although a fairly homogeneous sample was obtained for the present study in terms of gender, female caregivers still displayed a tendency towards higher burden compared to men. Previous studies suggest that both objective and subjective burden could modulate the perception of the stressful situation. Therefore, higher levels of burden could influence the assessment of the situation, leading to higher perceived stress [59,62]. In agreement with this hypothesis, our data showed a statistically significant positive correlation between perceived stress and burden in CCG. Those caregivers who displayed intense burden also reported higher levels of perceived stress. A statistically significant negative correlation between burden and resilience was also observed, in support of prior evidence [16]. This relationship can be mediated by social support, as previously reported in caregivers of people with dementia [63]. 

Results obtained in the present study should be interpreted with caution. Among the main limitations of the study is the limited size of the sample analyzed. Recruiting the sample, mainly from the group of caregivers, was particularly costly and difficult, since it is a very delicate sample that spends a lot of time caring for the oncologic patient (usually a relative) who in many cases suffers hospitalization. For this reason, the rate of abandonment in the study by caregivers was high. Another issue to consider for future studies is the use of standardized questionnaire batteries to measure variables such as the level of physical activity, social support, or leisure activities performed. In this case, these variables were obtained by means of the socio-demographic questionnaire developed by us, and it would be possible to obtain more significant results by using standardized instruments. Future studies will aim to increase the sample size, as well as the use of more specific measures of cognitive functioning. The possibility of exploring positive measures associated with care, such as satisfaction with care, which may be a modulating factor for the negative consequences observed in this study, should be also considered. A longitudinal approach in the case of caregivers (from the beginning of the care task), and taking into account the cancer stage of the oncologic patient, could also yield relevant and very interesting data on the effects of care at the cognitive and psychological level, as well as the modulating role of variables such as overload or perceived stress. Results obtained in this type of studies could offer us further scientific information in order to develop focused therapies for informal caregivers in order to improve the task of care minimizing negative impact of the burden frequently associated to this situation.

## 5. Conclusions

Results obtained suggest that informal caregivers of oncologic patients display burden, perceived stress, and subjective daily memory complaints. In addition, variables as time of diagnosis or prognosis could modulate their perceived stress. In general, an important percentage of caregivers that have participated in the present study have reported low levels of burden, however, those who displayed intense burden showed higher scores of subjective memory complaints and greater perceived stress than those who showed lack of burden. Therefore, the importance of a deep comprehensive assessment of caregivers is worth noting, as certain variables may be predictors of mental disorders. Further research is needed to evaluate the cognitive consequences of chronic exposure to cancer caregiving with the use of large sample sizes and longitudinal follow-ups. Thus, a better knowledge of the caregivers’ profile would allow more efficient early interventions tailored to meet the needs of both patient and caregiver [19,51].

Informal caregivers often have to make adaptations in their lifestyles in order to respond to the demands of the patient. Furthermore, caregivers must learn to plan for current and future needs, to cope with the process of their relative’s illness, with uncertainty and in the worst cases, with bereavement. For this reason, interventions intended to help reduce the physical, physiological, and psychosocial consequences of caregiving experienced both by patients and caregivers should be developed, assessed, and setup in order to promote their health [64].

## Figures and Tables

**Table 1:** Characteristics of caregivers and non-caregivers.

		CCG Group	Non-CG Group
**Sample (*N* = 60)**		34	26
Gender	Female	52.9%	61.5%
	Male	47.1%	38.5%
Age		50.09 ± 2.39	47.46 ± 2.15
Employment	Active	44.1%	65.4%
	Unemployed	29.4%	19.2%
	Medical leave	2.9%	3.8%
	Retired	23.5%	11.5%
Education level	No studies	2.9%	-
	Basic studies	41.2%	26.9%
	Medium grade studies	20.6%	23.1%
	Higher education	35.3%	50.0%
Relationship with cancer patient	Mother/Father	5.9%	-
	Sister/Brother	5.9%	-
	Daughter/Son	14.7%	-
	Partner	73.5%	-
Cohabitation with the family member	Live with cancer patient	88.2%	-
	Care at certain periods	2.9%	-
	Care more than 8 h/day	8.8%	-
	Live accompanied	-	84.6%
	Live on their own	-	15.4%
Care of the cancer patient	Unique caregiver	23.5%	-
	Several caregivers	26.5%	-
	Primary caregiver with support	14.7%	-
	Cohabitation with a self-sufficient patient	35.3%	-
Fatigue	Yes	41.2%	7.7%
	No	58.8%	92.3%
Sleep disturbances	Yes	41.2%	11.5%
	No	58.8%	88.5%
Physical activity	Yes	38.2%	61.5%
	No	61.8%	38.5%
Leisure activities	Yes	14.7%	38.5%
	No	85.3%	61.5%
Do you have someone to talk to?	Yes	67.6%	100.0%
	No	32.4%	0%
Do you have someone to distract yourself with?	Yes	50.0%	100.0%
	No	50.0%	0%

Abbreviations: CCG: Caregivers group; Non-CCG: Non-Caregivers group.

**Table 2:** Characteristics of cancer patients.

		CCG Group
**Sample (*N* = 60)**		34
Gender	Female	61.8%
	Male	38.2%
Time since diagnosis (months)	3–6 months	32.4%
	6–9 months	5.9%
	9–12 months	5.9%
	>12 months	55.9%
Prognosis	Favorable	58.8%
	Unfavorable	41.2%
Current treatment (not exclusive)	Chemotherapy	44.1%
	Radiotherapy	2.9%
	Other treatments	26.5%
	Without treatment	26.5%
Diagnosis (Type of cancer)	Breast	44.1%
	Lung	14.7%
	Non-Hodgkin lymphoma	2.9%
	Prostate	2.9%
	Osteosarcoma	2.9%
	Colon	14.7%
	Stomach	2.9%
	Myeloma	2.9%
	Ovary	5.9%
	Brain	2.9%
	Renal	2.9%

Abbreviations: CCG: Caregivers group.

**Table 3:** Correlations for the measures obtained in the caregivers’ group (CCG).

	MFE-30	CD-RISC-10	Rosenberg	PSS	**ZCBI**
**MFE-30** Pearson Correlation *p* N	1 34	−0.130 0.462 34	0.046 0.794 34	−0.017 0.925 0.343	−0.041 0.816 34
**CD-RISC-10** Pearson Correlation *p* N		1 34	0.410 * 0.016 34	−0.598 *** 0.000 34	−0.414 * 0.015 34
**ROSENBERG** Pearson Correlation *p* N			1 34	−0.502 ** 0.002 34	−0.320 0.065 34
**PSS** Pearson Correlation *p* N				1 34	0.492 ** 0.003 34
**ZCBI** Pearson Correlation *p* N					1 34

* *p* < 0.05; ** *p* > 0.01; *** *p* < 0.001; Abbreviations: CD-RISC-10: The Connor-Davidson Resilience Scale; MFE-30: Memory Failures of Everyday; PSS: Perceived Stress Scale; ROSENBERG: Rosenberg Self-Esteem Scale; ZCBI: Zarit Caregiver Burden Interview.
